# Serum mBDNF and ProBDNF Expression Levels as Diagnosis Clue for Early Stage Parkinson's Disease

**DOI:** 10.3389/fneur.2021.680765

**Published:** 2021-08-11

**Authors:** Xu Yi, Yujia Yang, Zhengfan Zhao, Manyu Xu, Yuan Zhang, Yingying Sheng, Junying Tian, Zhiqiang Xu

**Affiliations:** ^1^Department of Neurology and Center for Clinical Neuroscience, Daping Hospital, Third Military Medical University, Chongqing, China; ^2^Department of Foreign Language, Chongqing Medical University, Chongqing, China

**Keywords:** brain-derived growth factor, ProBDNF, Parkinson's disease, follow-up, diagnosis

## Abstract

Parkinson's disease (PD) is one of the most common chronic, progressive, and neurodegenerative diseases characterized clinically by resting tremor, bradykinesia, rigidity, and postural instability. As this disease is usually detected in the later stages, the cure is often delayed, ultimately leading to disability due to the lack of early diagnostic techniques. Therefore, it is of great importance to identify reliable biomarkers with high sensitivity and specificity for the early diagnosis of PD. In this study, we aimed to investigate whether serum expressions of mature brain-derived neurotrophic factor (mBDNF) and proBDNF can serve as biomarkers for the diagnosis of PD at early stage. One hundred and fifty-six patients with limb tremor and/or bradykinesia meeting the inclusion criteria were assigned to either ex-PD group (PD cases) or ex-NPD group (non-PD cases) and then reassigned to either po-PD group (with PD) or po-NPD group (without PD) at 1-year follow-up based on the results of the rediagnoses as performed in accordance with MDS Parkinson's diagnostic criteria. To improve early diagnostic accuracy, grouping (PD group and non-PD group) at initial visit and follow-up was performed differently and independently. Serum mBDNF and proBDNF levels were measured by enzyme-linked immunosorbent assays. The results demonstrated that serum levels of mBDNF and mBDNF/proBDNF were significantly lower in the ex-PD group (19.73 ± 7.31 and 0.09 ± 0.05 ng/ml) as compared with the ex-NPD group (23.47 ± 8.21 and 0.15 ± 0.12 ng/ml) (*p* < 0.01 for both) and in the po-PD group (19.24 ± 7.20 and 0.09 ± 0.05 ng/ml) as compared with the po-NPD group (25.05 ± 7.67 and 0.16 ± 0.14 ng/ml) (*p* < 0.01 for both). However, a significantly higher serum level of proBDNF was noted in the ex-PD group (235.49 ± 60.75 ng/ml) as compared with the ex-NPD group (191.75 ± 66.12 ng/ml) (*p* < 0.01) and in the po-PD group (235.56 ± 60.80 ng/ml) as compared with the po-NPD group (188.42 ± 65.08 ng/ml) (*p* < 0.01). In conclusion, mBDNF/proBDNF can be used as biomarkers for early stage Parkinson's disease; in addition, mBDNF plus proBDNF has better diagnostic value than mBDNF alone in the diagnosis of PD.

## Background

Parkinson's disease (PD) is a common chronic, progressive, and neurodegenerative condition characterized by the death of dopaminergic neurons in the substantia nigra, leading to progressive disabling, high morbidity, and heavy economic burden for both the patients and the society. Lewy body, a neuronal inclusion consisting largely of α-synuclein protein aggregations, is the pathologic hallmark of PD (1). As this disease is usually detected in the later stages, its cure is often delayed due to the lack of early diagnostic techniques (2). Thus, exploration of biomarkers is of great importance for the early detection of PD. In recent years, neuroscientists worldwide have shown an increasing interest in finding out diagnostic techniques for the early screening and accurate diagnosis of PD. To date, the diagnosis of PD still relies on clinical examination and follow-up (3), which has low accuracy for early diagnosis of PD. Therefore, it is of great significance to identify reliable biomarkers with high sensitivity and specificity for the early diagnosis of PD.

Mature brain-derived neurotrophic factor (mBDNF) has been shown to promote the growth and survival of synapses of dopamine neurons in the substantia nigra (4–6), and inhibition on mBDNF action leads to the loss of dopaminergic neurons (7–10). In a mouse model of PD, low expression of mBDNF has been shown to disrupt dopamine output in the corpus striatum, which is independent of dopaminergic expression in the substantia nigra (11–13). Patients with PD had low expression levels of mBDNF in both serum and substantia nigra (14–16), thus having an attenuated protective effect on the substantia nigra dopaminergic neurons (17). Interestingly, the increased expression of mBDNF level in the serum and cerebrospinal fluid in patients with progressive PD was associated with the use of anti-PD drugs (18–20). As the major component of Lewy bodies in PD, α-synuclein has been shown to effectively block the neurotrophic activity of mBDNF in the substantia nigra *via* downregulating mBDNF expression (11) and competitively inhibiting the mBDNF signaling at the receptor level (12). It has been reported that exogenous mBDNF is capable of reducing the loss of dopaminergic neurons in neuronal culture, and anti-Parkinson drugs like levodopa and exercise are beneficial to patients with PD by upregulating mBDNF expression (21, 22).

ProBDNF is the precursor of mBDNF and capable of converting to mBDNF by extracellular proteases. ProBDNF and mBDNF exert opposite effects by binding to p75 neurotrophin receptor (p75NTR) and tyrosine kinase receptor B (TkB) receptors, respectively. The imbalance in the ratio of mBDNF/proBDNF is associated with the pathogenesis of neuropsychiatric diseases. In neurodegenerative diseases, degenerated nerve cells can inhibit the conversion of proBDNF into mBDNF, resulting in an imbalance in the ratio of mBDNF/proBDNF. Notably, the above changes occur selectively in the substantia nigra pars compact, striatum, and hippocampus (23, 24) and are remarkably associated with the severity of PD (25). mBDNF and proBDNF that are mainly produced or released from the brain can be derived from the peripheral and central nervous systems. Both proBDNF and mBDNF can pass through the blood–brain barrier freely; thus, their levels in the serum could be regarded as their actual levels in the central nervous system (26). It has been demonstrated that the change in serum levels of proBDNF and mBDNF are implicated in neurodegenerative diseases, including AD, Huntington, and schizophrenia (27–29). However, it remains unclear whether the change in serum levels of proBDNF and mBDNF are correlated with early PD and whether they can serve as biomarkers for early PD diagnosis.

In this study, we aimed to investigate the association of serum levels of proBDNF and mBDNF with the diagnosis of PD through serum tests on proBDNF and mBDNF levels in a clinical cohort of newly diagnosed PD cases. Since follow-up is an important means to improve the accuracy of PD diagnosis, we re-evaluated the PD diagnosis of all cases, made corrections for the misdiagnosis at 1-year follow-up visit, and reanalyze the correlation between the biochemical parameters and the PD diagnosis at 1-year follow-up. Furthermore, the PD diagnoses before and after follow-up were assessed comparatively for investigation on the significance of proBDNF, mBDNF, and mBDNF/proBDNF ratio in the early diagnosis of PD.

## Materials and Methods

### Study Subjects

This study was conducted in patients with dyspraxia admitted to the Department of Neurology of Daping Hospital between January 2015 and December 2018. The eligible subjects should be those with initial diagnosis of Parkinson's syndrome characterized by limb tremor or bradykinesia, onset duration of <1 year and Hoehn–Yahr grade of <2.5, and willingness to participate in this study and undergo blood biomarker tests. Those patients were ineligible if they had movement disorders, including fractures, strokes, spinal cord lesions, abnormal thyroid function, electrolyte disorders, cardiopulmonary insufficiency, cognitive impairment, and mental disorders. A total of 156 patients who met the Movement Disorder Society (MDS) Parkinson's disease diagnostic criteria (30) were assigned to undergo clinical evaluation including inquiry in medical history and physical and laboratory examination. None of these patients had a history of administration of anti-Parkinson drugs and antidepressant drugs at baseline. This study was approved by the Institutional Review Board of Daping Hospital.

### Clinical Assessments

The patients with dyskinesia were divided into the ex-PD group (those with PD) and ex-NPD group (those without PD) based on their diagnosis on first visit in accordance with MDS Parkinson's diagnostic criteria (30). All patients were followed up for 1 year. The initial PD diagnosis was re-evaluated by two PD specialists based on the natural changes of patients in clinical symptoms and their responses to dopamine-like drugs. Based on the follow-up evaluation on PD diagnosis, the patients were reassigned to either the po-PD group (with PD) or the po-NPD group (without PD).

### Measurements of Serum Levels of proBDNF and mBDNF

The blood samples of the included PD patients were collected at 7:00–9:00 a.m. at initial visit (because blood sampling done then was free of interference from circadian rhythm). Fasting blood samples were centrifuged at 3,000×*g* after 60 min of incubation. The supernatant was taken for ELISA assay. The levels of human mBDNF and proBDNF in the serum were measured using Mature BDNF Rapid ELISA Kit and proBDNF Rapid ELISA Kit (Biosensis, Thebarton, Australia) according to the instructions of the manufacturer. First, add mature BDNF or proBDNF standards and samples to the precoated microplate wells and incubate for 45 min. Then, discard the solution inside the wells and perform five washes. Add detection antibody into each well and incubate for 30 min. Discard and wash as described above. Add the 1 × streptavidin–HRP conjugate into each well for 30 min. Discard and wash as described above again. Finally, add TMB and stop solution according to the instructions. Absorbance values for each sample were read at 450 nm on a plate reader (31).

### Statistical Analysis

The statistical significance of differences between groups was analyzed by the two-sample independent *t*-test, the Mann–Whitney *U*-test, the chi-squared test, Fisher's exact test, or analysis of variance (ANOVA) according to the characteristics of the data. The data were expressed either as the mean ± standard deviation (SD) for numerical variables or as count (%) for categorical variables. Confidence intervals (CIs) at the 95% level were calculated for the odds ratios (ORs). ROC curves were analyzed to evaluate the capacity of biomarkers in discriminating between PD and NPD cases. The optimum cutoff values for each biomarker were determined using the highest Youden's index (sensitivity + specificity − 1). All hypothesis tests were two-sided, and statistical significance was defined as *p* < 0.05. All statistical computations were performed using SPSS version 19.0 (SPSS Inc., Chicago, IL, USA), and all figures were created using a graphics package (GraphPad Prism, version 6).

## Results

### Baseline Data of Newly Diagnosed Patients

A total of 156 newly diagnosed patients with limb tremor and/or bradykinesia were assigned to the ex-PD group (*n* = 111) and the ex-NPD group (*n* = 45) according to MDS Parkinson's disease diagnostic criteria. As summarized in Table 1, there were no significant differences in age, gender, hypertension, diabetes, hyperlipidemia, and years of education between the ex-PD group and the ex-NPD group (*p* > 0.05) (Table 1).

**Table 1:** Baseline data of newly diagnosed patients.

**Variables**	**Ex-PD**	**Ex-NPD**	***t*/*x*^**2**^/*z***	***p-*value**
Sample size	111	45		
Age, years, mean ± SD	61.1 ± 8.8	60.0 ± 9.2	−0.728	0.467^a^
Male, *n* (%)	65 (58.6)	21 (46.7)	1.381	0.176^b^
Education years, median (IQR)	8.0 (5.0–12.0)	9.0 (7.0–13.0)	1.303	0.192^c^
Hypertension, *n* (%)	9 (8.1)	6 (13.3)	0.495	0.482^b^
Diabetes, *n* (%)	8 (7.2)	5 (11.1)	0.230	0.632^b^
Hyperlipidemia, *n* (%)	31 (27.9)	16 (35.6)	0.885	0.347^b^
Exercise, *n* (%)	16 (14.4)	5 (11.1)	0.300	0.584^b^

a *Student's t-test*.

b *Chi-squared test*.

c *Mann–Whitney U-test*.

### Comparison of Serum Levels of proBDNF, mBDNF, and mBDNF/proBDNF Between the Ex-PD Group and the Ex-NPD Group

Serum proBDNF levels were significantly higher in the ex-PD group than in the ex-NPD group (235.49 ± 60.75 vs. 191.75 ± 66.12 ng/ml, *t* = −3.970, *df* = 154, *p* = 0.0001). However, the serum levels of mBDNF and mBDNF/proBDNF ratio were significantly lower in the ex-PD group than in the ex-NPD group (19.73 ± 7.31 vs. 23.47 ± 8.21 ng/ml for mBDNF, *t* = 2.794, *df* = 154, *p* = 0.0059; 0.09 ± 0.05 vs. 0.15 ± 0.12 for mBDNF/proBDNF, *t* = 4.216, *df* = 154, *p* < 0.0001).

### Baseline Data at 1-Year Follow-Up Visit

All of the included patients were followed up for 1 year. In accordance with the MDS Parkinson's disease diagnostic criteria, the included patients were re-evaluated for PD diagnosis based on their clinical characteristics including the changes in clinical symptoms after receiving antiparkinsonian medication. Six cases in the ex-PD group were redefined as not having PD, while nine cases in the ex-NPD group were diagnosed with PD in the follow-up. Based on the new diagnosis, the patients were redivided into po-PD group (PD patients, *n* = 114) and po-NPD group (non-PD patients, *n* = 42). As indicated in Table 2, there were no significant differences in age, gender, hypertension, diabetes, hyperlipidemia, and years of education between the po-PD group and the po-NPD group (*p* > 0.05).

**Table 2:** Baseline data after 1-year follow-up.

**Variables**	**Po-PD**	**Po-NPD**	***t*/*x*^**2**^/*z***	***p-*value**
Sample size	114	42		
Age, years, mean ± SD	61.5 ± 8.6	58.9 ± 9.7	−1.652	0.099^a^
Male, *n* (%)	62 (54.4)	24 (57.1)	0.094	0.759^b^
Education years, median (IQR)	9.0 (6.0–12.0)	9.0 (6.0–12.3)	0.230	0.257^c^
Hypertension, *n* (%)	9 (7.9)	6 (14.3)	0.801	0.371^b^
Diabetes, *n* (%)	8 (7.0)	5 (11.9)	0.427	0.514^b^
Hyperlipidemia, *n* (%)	34 (29.8)	13 (31.0)	0.019	0.892^b^
Exercise, *n* (%)	16 (14.0)	5 (11.9)	0.120	0.730^b^

a *Student's t-test*.

b *Chi-squared test*.

c *Mann–Whitney U-test*.

### Comparison of Serum Levels of proBDNF, mBDNF, and mBDNF/proBDNF Between the po-PD Group and the po-NPD Group

Biochemical analyses showed that serum levels of mBDNF and mBDNF/proBDNF in the po-PD group were significantly lower than in the po-NPD group (19.24 ± 7.20 vs. 25.05 ± 7.67 ng/ml for mBDNF, *t* = 4.392, *df* = 154, *p* < 0.0001; 0.09 ± 0.05 vs. 0.16 ± 0.14 for mBDNF/proBDNF, *t* = 5.179, *df* = 154, *p* < 0.0001). However, serum proBDNF levels were significantly higher in the po-PD group than in the po-NPD group (235.56 ± 60.80 vs. 188.42 ± 65.08 ng/ml, *t* = −4.214, *df* = 154, *p* < 0.0001).

### Longitudinal Comparison of the Levels of proBDNF, mBDNF, and mBDNF/proBDNF Between the Ex-PD Group and the Po-PD Group

The ROC curve was utilized to evaluate the association of proBDNF, mBDNF, and mBDNF/proBDNF with the diagnosis of PD. The results showed that there were no significant differences in the area under the curve (AUC) of proBDNF, mBDNF, and mBDNF/proBDNF between the ex-PD and po-PD groups (0.673 vs. 0.711, *p* = 0.57; 0.632 vs. 0.71, *p* = 0.26; 0.716 vs. 0.781, *p* = 0.28, respectively) (Table 3).

**Table 3:** Diagnostic performance of the mBDNF, proBDNF, and mBDNF/proBDNF.

	**Ex**			**Po**		
	**mBDNF**	**ProBDNF**	**mBDNF/proBDNF**	**mBDNF**	**ProBDNF**	**mBDNF/proBDNF**
AUC	0.632 (0.533–0.731)	0.673 (0.578–0.768)	0.716 (0.629–0.804)	0.712 (0.621–0.802)	0.644 (0.542–0.746)	0.749 (0.666–0.832)
Cutoff value	24.66	175.7	0.1171	24.19	220.8	0.1171
Sensitivity, %	75.7	85.6	80.2	76.3	63.2	80.7
Specificity, %	48.9	44.4	55.6	57.1	64.3	59.5
PPV, %	78.5	79.2	81.7	82.9	82.8	84.4
NPV, %	44.9	55.6	53.2	47.1	39.1	53.2

*PPV, positive predictive value; NPV, negative predictive value*.

### Horizontal Comparison of the Levels of proBDNF, mBDNF, and mBDNF/proBDNF Ratio Between the Two Groups at Initial Visit and Follow-Up Visit

**Figure 1:**
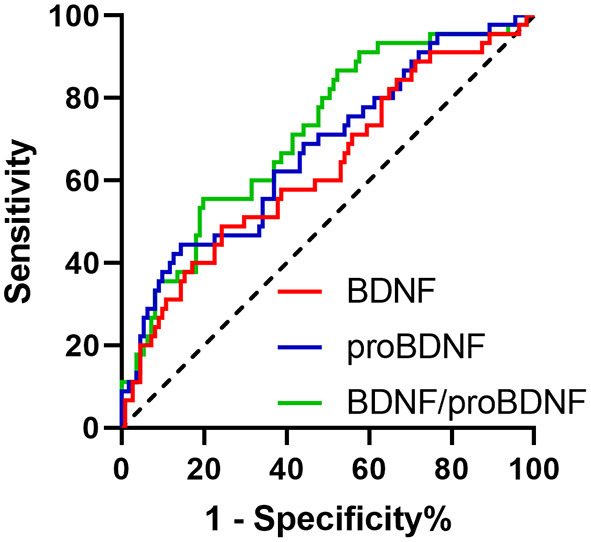
ROC curves of proBDNF, mBDNF, and mBDNF/proBDNF in the initial diagnosis of PD.

In the initial visit, the AUC of serum proBDNF, mBDNF, and mBDNF/proBDNF ratio stood at 0.673 (SE: 0.0486, CI: 0.593–0.746), 0.632 (SE: 0.0508, CI: 0.551–0.708), and 0.716 (SE: 0.0450, CI: 0.639–0.786), respectively. As shown in Figure 1, there was a statistically significant difference in the AUC between mBDNF and mBDNF/proBDNF ratio (*p* = 0.0194).

**Figure 2:**
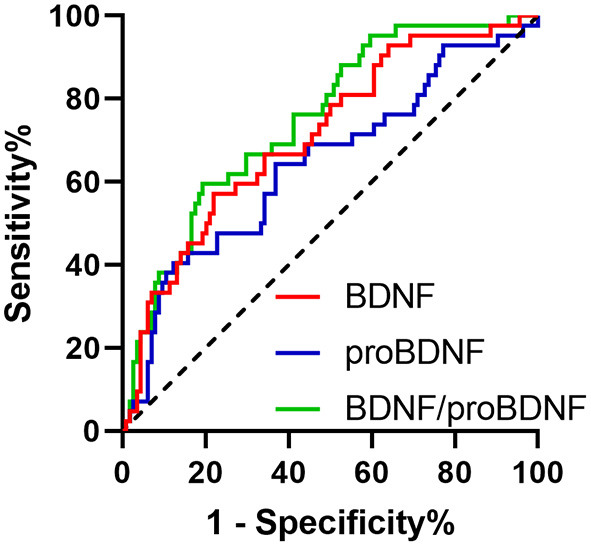
ROC curves of proBDNF, mBDNF, and mBDNF/proBDNF in the follow-up diagnosis of PD.

At 1-year follow-up, the AUC of serum proBDNF, mBDNF, and mBDNF/proBDNF ratio stood at 0.695 (SE: 0.0493, CI: 0.616–0.766), 0.710 (SE: 0.0476, CI: 0.632–0.780), and 0.781 (SE: 0.0400, CI: 0.708–0.843), respectively. As depicted in Figure 2, there was a significant difference in the AUC between mBDNF and mBDNF/proBDNF ratio (*p* = 0.0489).

## Discussion

According to the UK Parkinson's Disease Brain Bank diagnostic criteria, continuous clinical follow-up could be an important means to improve the accuracy of PD diagnosis (30). In our study, some initially suspected PD cases/patients were confirmed as non-PD cases in the follow-up visit. The change of the PD diagnosis for some patients occurred during follow-up that might influence the correlation between these biomarkers and the PD diagnoses; thus, we reassessed the correlation between these biomarkers and the more accurate diagnoses of PD at follow-up and compared the differences between the initial diagnoses and the follow-up diagnoses in the value of these biomarkers.

In our study, the serum level of mBDNF remained significantly lower while that of proBDNF remained significantly higher in patients with early PD than in the control subjects even when the diagnosis of PD has been corrected at follow-up, indicating the reliability of mBDNF as a biomarker for PD. Our finding that serum mBDNF level in patients with early PD was significantly lower than in the healthy population is in line with literature (31–34). It has been suggested that the reduction in serum mBDNF level is associated with attenuation in both the protective effect of mBDNF on the survival and functioning of substantia nigra dopamine neurons and the maintenance of neuronal function, thereby facilitating the pathological changes of PD (19, 35). mBDNF that was mainly produced and released from the brain has been shown to penetrate the blood–brain barrier freely; thus, BDNF levels measured in the periphery reflect mBDNF brain levels (26). In contrast to a previous study in which healthy subjects were used as control, in our study, the control subjects were non-Parkinson patients who visited the doctor with chief complaints of tremor and bradykinesia, indicating the potential value of the serum mBDNF level in differentiating PD from Parkinson's syndrome.

Our study also shows that the level of proBDNF remained significantly higher in early PD patients than in the control group at 1-year follow-up, which might be attributed to the inhibition of the transformation of proBDNF into mBDNF in PD patients (24). ProBDNF has been shown to bind to p75NTR with high affinity to mediate apoptosis and promote long-term inhibition (23). Previous studies suggested the close association between proBDNF level changes and the pathological changes of PD, whereas our study showed the correlation between proBDNF level and the clinical diagnosis of PD, suggesting that proBDNF might serve as one of the clues for the diagnosis of PD.

The application of mBDNF/proBDNF was found to result in greater variation in terms of decrease of serum mBDNF level and increase in serum proBDNF level. The values of serum levels of mBDNF, mBDNF, and mBDNF/proBDNF in the diagnosis of PD were evaluated using ROC curves, with comparison made both transversely and longitudinally. In cross-sectional comparison, mBDNF/proBDNF showed stronger correlation with PD diagnosis than did serum mBDNF level either at initial diagnosis or follow-up, with differences statistically significant, suggesting that mBDNF/proBDNF as one of the diagnosis clues for PD might be better than mBDNF and proBDNF alone.

In this study, we investigated the association of proBDNF, mBDNF, or mBDNF/proBDNF ratio with early diagnosis of PD in patients in Chongqing, China. In order to improve the diagnostic accuracy of PD at follow-up visit and obtain more convincing conclusions, we employed a more reliable diagnostic technique in follow-up to evaluate the significance of early biomarkers in the diagnosis of PD. However, these findings remain to be validated further in a future larger cohort study. However, there are limitations in our study; for example, the sample size is limited and the effect of the body mass index has not been considered.

## Conclusions

In conclusion, serum mBDNF/proBDNF ratio has better diagnostic value than mBDNF or proBDNF alone in the diagnosis of PD. Therefore, in order to enhance the accuracy of PD diagnosis, a test of serum mBDNF/proBDNF is valuable, especially at the early stage of Parkinson's disease.

## Data Availability Statement

The raw data supporting the conclusions of this article will be made available by the authors, without undue reservation.

## Ethics Statement

The studies involving human participants were reviewed and approved by the Institutional Review Board of Daping Hospital. The patients/participants provided their written informed consent to participate in this study.

## Author Contributions

XY and ZX conceived and designed the study. ZZ and MX performed the experiments. YZ and YS collected patient data. XY and YY wrote the paper. ZX and JT reviewed and edited the manuscript. All authors read and approved the manuscript.

## Conflict of Interest

The authors declare that the research was conducted in the absence of any commercial or financial relationships that could be construed as a potential conflict of interest.

## Publisher's Note

All claims expressed in this article are solely those of the authors and do not necessarily represent those of their affiliated organizations, or those of the publisher, the editors and the reviewers. Any product that may be evaluated in this article, or claim that may be made by its manufacturer, is not guaranteed or endorsed by the publisher.
